# Out-of-unison resonance in weakly nonlinear coupled oscillators

**DOI:** 10.1098/rspa.2014.0659

**Published:** 2015-01-08

**Authors:** T. L. Hill, A. Cammarano, S. A. Neild, D. J. Wagg

**Affiliations:** 1 Department of Mechanical Engineering, University of Bristol, Bristol BS8 1TR, UK; 2 Department of Mechanical Engineering, University of Sheffield, Sheffield S1 3JD, UK

**Keywords:** nonlinear oscillator, normal form, internal resonance, backbone curve

## Abstract

Resonance is an important phenomenon in vibrating systems and, in systems of nonlinear coupled oscillators, resonant interactions can occur between constituent parts of the system. In this paper, out-of-unison resonance is defined as a solution in which components of the response are 90° out-of-phase, in contrast to the in-unison responses that are normally considered. A well-known physical example of this is whirling, which can occur in a taut cable. Here, we use a normal form technique to obtain time-independent functions known as backbone curves. Considering a model of a cable, this approach is used to identify out-of-unison resonance and it is demonstrated that this corresponds to whirling. We then show how out-of-unison resonance can occur in other two degree-of-freedom nonlinear oscillators. Specifically, an in-line oscillator consisting of two masses connected by nonlinear springs—a type of system where out-of-unison resonance has not previously been identified—is shown to have specific parameter regions where out-of-unison resonance can occur. Finally, we demonstrate how the backbone curve analysis can be used to predict the responses of forced systems.

## 1. Introduction

Understanding resonant interactions in nonlinear systems is a crucial step in understanding their global dynamic behaviour. A large class of resonant interactions may be described as a vibration-in-unison, meaning that the physical displacement coordinates of the system reach their extrema and pass through zero simultaneously. The vibration-in-unison concept was famously used by Rosenberg to give the first definition of a nonlinear normal mode for a conservative system [1–4]. In more recent work, the definition of nonlinear normal modes has been relaxed to include more general periodic motions (e.g. [5–9]). In this paper, we investigate the opposite case to that defined by Rosenberg, namely out-of-unison resonances, and demonstrate how they manifest themselves in systems of weakly nonlinear coupled oscillators.

Here we define out-of-unison resonance as a resonant interaction between two displacement coordinates of a conservative system, where one coordinate reaches an extrema as the other passes through zero, such that they are 90° out-of-phase. The best-known physical example of this behaviour is whirling which can occur, for example, in a taut cable [10]. As we shall show later, this type of resonance occurs when two underlying linear natural frequencies of the system are close, giving a resonance at a 1 : 1 frequency ratio.

Previous authors have applied approximate analytical techniques to problems involving weakly nonlinear oscillators (e.g. [11–15]). The phenomenon of internal resonance has also been analysed using similar approaches (for example [16] and references therein). An alternative method for studying internal resonance is to use nonlinear normal modes, as discussed in [7,17–22]. These techniques use an energy-based representation to show the free vibration responses of nonlinear systems.

In this paper, the second-order normal form technique [23] is used to analytically describe the dynamics of weakly nonlinear, conservative systems. These dynamical behaviours are equivalent to the nonlinear normal modes of the systems; however, they are represented in terms of displacement amplitudes rather than energy. As such, this technique is arguably better suited for practical applications. Furthermore, as this technique is applied directly to the second-order differential equations (the conventional formulation of many vibration problems), it lends itself to such problems more naturally than the classical first-order, state-space, equivalent [24–27]. A comparison between the first- and second-order normal form technique is given in [23], and further details on the second-order normal form technique can be found in [28–30].

A method for the application of the second-order normal form technique is introduced in §2. Then, in §3, we consider whirling motion in a taut, horizontal, undamped cable, which is a well-known physical example of out-of-unison resonance. In §4, we consider an in-line, conservative, two degree-of-freedom (2-d.f.) oscillator to demonstrate how out-of-unison motion may exist in other nonlinear systems. Furthermore, we demonstrate how, from the analytical descriptions developed, we may determine the values of the physical parameters that yield out-of-unison resonance. Finally, we demonstrate how the out-of-unison motion, seen in the conservative systems, translates to a similar behaviour when the in-line system is forced and damped (and so is non-conservative).

## 2. Second-order normal form technique

In this paper, we use the second-order normal form technique to transform the equations of motion of weakly nonlinear systems into sets of time-invariant equations, describing the approximate dynamics. The technique uses the assumption that the nonlinear terms are small; therefore, as the amplitude decreases the results converge to the true solution, but they diverge at higher amplitudes. Here, we consider responses in amplitude and frequency ranges where the nonlinear terms are small, and it is confirmed that the resulting errors are negligible via comparison with results found using numerical continuation. Methods for directly estimating the validity of the normal form results may also be used (e.g. [31]). The derivation and generalized application of the technique are given in [23,28], and the approach used here is also shown in [30]. For completeness, we now outline the key steps of the second-order normal form technique that are applied to the systems considered in this paper.

The equation of motion for a system with *N* d.f. may be written

2.1 $$\text{M}\ddot{\text{x}}+\text{C}\dot{\text{x}}+\text{K}\text{x}+{\text{N}}_{x}(\text{x})={\text{P}}_{x}\cos(\Omega_{f}t),$$

where **M**, **C** and **K** are the {*N*×*N*} mass, damping and stiffness matrices respectively, **N**_*x*_ and **P**_*x*_ are {*N*×1} vectors of nonlinear terms and forcing amplitudes, respectively, and *Ω*_*f*_ is the forcing frequency. To find the backbone curves of this system, we consider the unforced and undamped equivalent of equation (2.1), which may be written as follows:

2.2 $$\text{M}\ddot{\text{x}}+\text{K}\text{x}+{\text{N}}_{x}(\text{x})=0.$$

We now apply the linear modal transform **x**=*Φ***q**, where *Φ* is an {*N*×*N*} matrix whose *n*th column describes the modeshape of the *n*th linear mode, and **q** is an {*N*×1} vector of linear modal displacements. This allows equation (2.2) to be written as follows:

2.3 $$\ddot{\text{q}}+\Lambda \;\Lambda \text{q}+{\text{N}}_{q}(\text{q})=0,$$

where *Λ* is an {*N*×*N*} diagonal vector where the *n*th leading diagonal element is the square of the *n*th natural frequency of the underlying linear system, $\omega_{nn}^{2}$, and **N**_*q*_ is an {*N*×1} vector of nonlinear terms.

Next we apply the nonlinear near-identity transform **q**=**u**+**h**(**u**), where **u** describes the fundamental response, and **h** (which is assumed to be small) contains the harmonic contents of **q**. From this, we can write the transformed equation of motion as

2.4 $$\ddot{\text{u}}+\Lambda \;\Lambda \text{u}+{\text{N}}_{u}(\text{u})=0.$$

We assume *u*_*n*_ (the *n*th element of **u** and the fundamental response of *q*_*n*_) to be sinusoidal, such that it may be written

2.5 $$u_{n}=u_{np}+u_{nm}=\frac{U_{n}}{2}\;e^{+j(\omega_{rn}t-\phi_{n})}+\frac{U_{n}}{2}\;e^{-j(\omega_{rn}t-\phi_{n})},$$

where *U*_*n*_, *ω*_*rn*_ and *ϕ*_*n*_ are the amplitude, response frequency and the phase of *u*_*n*_, respectively. Note that *ω*_*rn*_ and *ω*_*nn*_ are distinct and represent the fundamental response frequency and linear natural frequency of *q*_*n*_, respectively. The subscripts *p* and *m* denote the positive and negative (plus and minus) signs of the complex exponents, respectively.

As we are assuming the system to be weakly nonlinear, we assume that **N**_*q*_ is small in relation to other terms—denoted as order *ɛ*^1^, where *ɛ* is a bookkeeping parameter denoting *smallness*. Furthermore, as the harmonics are small we may also state that **h** is of order *ɛ*^1^. Therefore, we may write *ɛ***N**_*q*_(**u**+*ɛ***h**)≈*ɛ***N**_*q*_(**u**) whilst maintaining order *ɛ*^1^ accuracy. Substituting equation (2.5) into **N**_*q*_(**u**) allows us to write **N**_*q*_(**u**)=[*n*_*q*_]u* where [*n*_*q*_] is an {*N*×*L*} matrix of coefficients and u* is an {*L*×1} vector of all the unique combinations of *u*_*np*_ and *u*_*nm*_ that exist in **N**_*q*_(**u**). The ℓth element of u* can be written as follows:

2.6 $$u_{\ell }^{*}=\prod_{k=1}^{N}\{u_{kp}^{s_{\ell kp}}u_{km}^{s_{\ell km}}\},$$

where *s*_ℓ*kp*_ and *s*_ℓ*km*_ are the exponents of *u*_*kp*_ and *u*_*km*_ in $u_{\ell }^{*}$, respectively.

Substituting equation (2.5) into equation (2.6) leads to

2.7 $$u_{\ell }^{*}=\left[\prod_{k=1}^{N}{\left(\frac{U_{k}}{2}\right)}^{\left(s_{\ell kp}+s_{\ell km}\right)}\right]e^{j({\bar{\omega }}_{\ell }t-{\bar{\phi }}_{\ell })},$$

where ${\bar{\omega }}_{\ell }$ and ${\bar{\phi }}_{\ell }$ are the response frequency and phase of $u_{\ell }^{*}$, respectively, and may be found using

2.8 $${\bar{\omega }}_{\ell }=\sum_{k=1}^{N}\{(s_{\ell kp}-s_{\ell km})\omega_{rk}\}\;\mathrm{and}\;{\bar{\phi }}_{\ell }=\sum_{k=1}^{N}\{(s_{\ell kp}-s_{\ell km})\phi_{k}\}.$$

We now define the {*N*×*L*} matrix *β*, where element {*n*, ℓ} of *β* is given by

2.9 $$\beta_{n,\ell }={\bar{\omega }}_{\ell }^{2}-\omega_{rn}^{2}={\left[\sum_{k=1}^{N}\{(s_{\ell kp}-s_{\ell km})\omega_{rk}\}\right]}^{2}-\omega_{rn}^{2}.$$

Therefore, if the response frequency of $u_{\ell }^{*}$, ${\bar{\omega }}_{\ell }$, is equal in magnitude to that of the *n*th linear mode, *ω*_*rn*_, then *β*_*n*,ℓ_=0. Hence any element in *β* with a value of zero corresponds to an element in [*n*_*q*_] describing the coefficient of a resonant term. We can now find the resonant nonlinear terms (see equation (2.4)) by defining **N**_*u*_=[*n*_*u*_]u*, where [*n*_*u*_] is an {*N*×*L*} matrix of coefficients. As [*n*_*u*_] must be populated by the coefficients of resonant terms we use, for the {*n*,ℓ}th term

2.10a $${\left[n_{u}\right]}_{n,\ell }={\left[n_{q}\right]}_{n,\ell }\;\text{if:}\;\beta_{n,\ell }=0$$

and

2.10b $${\left[n_{u}\right]}_{n,\ell }=0\;\;\;\text{if:}\;\beta_{n,\ell }\neq 0.$$

Although the calculation of **h** is not explicitly needed here, it may be found using **h**=[*h*]u* where *h*_*n*,ℓ_=*n*_*q*,*n*,ℓ_/*β*_*n*,ℓ_ when *β*_*n*,ℓ_≠0, and *h*_*n*,ℓ_=0 when *β*_*n*,ℓ_=0 (see [23] for further details).

As all terms in the *n*th element of the vector **N**_*u*_, written *N*_*u*,*n*_, resonate at frequency *ω*_*rn*_, we may write

2.11 $$N_{u,n}=N_{u,n}^{+}\;e^{+j\omega_{rn}t}+N_{u,n}^{-}\;e^{-j\omega_{rn}t},$$

where $N_{u,n}^{+}$ and $N_{u,n}^{-}$ are complex conjugates. Substituting equations (2.5) and (2.11) into equation (2.4) allows us to write

2.12 $$\left[(\omega_{nn}^{2}-\omega_{rn}^{2})\frac{U_{n}}{2}\;e^{-j\phi_{n}}+N_{u,n}^{+}\right]e^{+j\omega_{rn}t}+\left[(\omega_{nn}^{2}-\omega_{rn}^{2})\frac{U_{n}}{2}\;e^{+j\phi_{n}}+N_{u,n}^{-}\right]e^{-j\omega_{rn}t}=0.$$

From equation (2.12), we can see that the expressions in the square brackets are a complex conjugate pair. Therefore, as both of these expressions must equal zero, we may write the first expression as

2.13 $$(\omega_{nn}^{2}-\omega_{rn}^{2})U_{n}+2N_{u,n}^{+}\;e^{+j\phi_{n}}=0.$$

This allows us to write *N* equations in the form of equation (2.13), which may be solved to find all *U*_*n*_ and *ϕ*_*n*_. In the cases considered here we assume the harmonics to be negligible and so we can write **q**=**u**, such that the linear modal transform may be written **x**=*Φ***u**.

## 3. An example cable system

**Figure 1.**
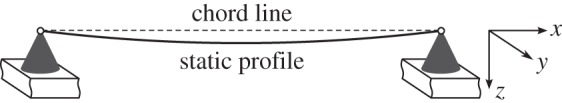
A diagram of an unforced, undamped, horizontal cable. The physical coordinates *x*, *y* and *z* are defined, where *x* is in the direction of the chord line and *z* is the direction of gravity.

Consider the dynamics of an unforced, undamped, horizontal cable under tension, as represented in figure 1. The dynamics of the cable can be described using the modal equations of motion derived by Warnitchai et al. [32], where the system is modelled using the sets of linear modes of the cable in the *y*- and *z*-directions. It is assumed that the motion in the *x*-direction is negligible. Owing to the influence of gravity (which acts in the *z*-direction), the cable exhibits a small sag, breaking the rotational symmetry about *x*. This leads to a difference in the dynamic behaviour of the corresponding linear modes in the *y*-direction and in the *z*-direction—most notably, a difference in the linear natural frequencies [14]. Here we consider the responses of the cable in the vicinity of the natural frequencies of the first linear modes in the *y*- and *z*-directions, whose displacements are written as *q*_1_ and *q*_2_, respectively. These two modes are used to describe the dynamics that are of particular interest.

**Figure 2.**
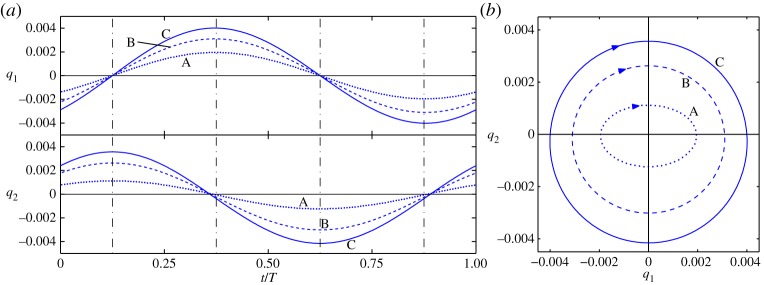
Three whirling responses of a cable, giving examples of out-of-unison motion. The relevant parameter values are *ω*_*n*1_=122.04 rad s^−1^, *ω*_*n*2_=123.87 rad s^−1^ and *W*=3.2×10^8^ m^−2^ s^−2^. The dotted, dashed and solid lines show responses A, B and C, respectively (corresponding to three responses in figure 3). Panel (*a*) is in the projection of normalized time, *t*/*T* (where *T* is the period), against the linear modal coordinates, *q*_1_ and *q*_2_ in the top and bottom axes, respectively. Panel (*b*) is in the projection of *q*_1_ against *q*_2_, with arrows illustrating motion in the clockwise direction. (Online version in colour.)

An example of out-of-unison motion between coordinates can be seen in figure 2, which shows three whirling responses for the cable—labelled A, B and C. Figure 2*a* shows the projection of normalized time, *t*/*T* (where *T* is the period), against *q*_1_ and *q*_2_. Figure 2*b* shows the projection *q*_1_ against *q*_2_, the responses of the first linear modes in the horizontal and vertical directions, respectively, parametrized in time. As such, figure 2*b* is analogous to the path of motion of the cable in the *y*–*z* plane. It can clearly be seen that these coordinates are not vibrating in-unison. Instead, as one coordinate reaches an extrema, the other is passing through zero (although a slight deviation is seen, due to the sagging of the cable under gravity). As this system is conservative, this out-of-unison motion is not due to the influence of forcing or damping, but rather is a fundamental underlying behaviour of the system.

### (a) Normal form decomposition

Using the normal form technique described in §2, we investigate the mechanisms behind the out-of-unison motion of the cable seen in figure 2. As the Warnitchai formulae describe the motion of the cable using modal coordinates, the equations of motion may be written in the form of equation (2.3) directly, where

3.1 $$\text{q}=\left(\begin{array}{c} q_{1}\\ q_{2} \end{array}\right),\;\Lambda \;\Lambda =\left[\begin{array}{cc} \omega_{n1}^{2} & 0\\ 0 & \omega_{n2}^{2} \end{array}\right]\;\mathrm{and}\;{\text{N}}_{q}=\left(\begin{array}{c} W(q_{1}^{3}+q_{1}q_{2}^{2})+2Q(q_{1}q_{2})\\ W(q_{2}^{3}+q_{1}^{2}q_{2})+Q(q_{1}^{2}+3q_{2}^{2}) \end{array}\right),$$

where *ω*_*n*1_ and *ω*_*n*2_ (rad s^−1^) are the linear natural frequencies of the first linear modes in the *y*- and *z*-directions, respectively, and the nonlinear parameters are calculated using *W*=*ν*_11_/*m* and *Q*=*β*_11_/*m*. The parameter *ν*_11_ (kg m^−2^ s^−2^) arises from the stretching of the cable under deformation, *β*_11_ (kg m^−1^ s^−2^) describes the effect of the static sag and *m* (kg) is the mass of the cable. Details regarding how *ω*_*n*1_, *ω*_*n*2_, *ν*_11_ and *β*_11_ are calculated are given in [32].

Internally resonant responses require an integer ratio between the fundamental response frequencies, *ω*_*r*1_ and *ω*_*r*2_. As we are interested in internally resonant responses in the vicinity of the linear natural frequencies, which are close, we shall consider the case where *Ω*=*ω*_*r*1_=*ω*_*r*2_, where *Ω* denotes the common response frequency. Making the substitution *q*_*n*_=*u*_*np*_+*u*_*nm*_ into **N**_*q*_ (see equation (2.5)) we calculate [*n*_*q*_], u* and *β* as

3.2 $${\left[n_{q}\right]}^{T}=\left[\begin{array}{cc} W & 0\\ 3W & 0\\ 3W & 0\\ W & 0\\ 2W & 0\\ W & 0\\ W & 0\\ W & 0\\ 2W & 0\\ W & 0\\ 2Q & 0\\ 2Q & 0\\ 2Q & 0\\ 2Q & 0\\ 0 & W\\ 0 & W\\ 0 & 2W\\ 0 & 2W\\ 0 & W\\ 0 & W\\ 0 & W\\ 0 & 3W\\ 0 & 3W\\ 0 & W\\ 0 & Q\\ 0 & 2Q\\ 0 & Q\\ 0 & 3Q\\ 0 & 6Q\\ 0 & 3Q \end{array}\right],\;{\text{u}}^{*}=\left[\begin{array}{c} u_{1p}^{3}\\ u_{1p}^{2}u_{1m}\\ u_{1p}u_{1m}^{2}\\ u_{1p}u_{2p}^{2}\\ u_{1p}u_{2p}u_{2m}\\ u_{1p}u_{2m}^{2}\\ u_{1m}^{3}\\ u_{1m}u_{2p}^{2}\\ u_{1m}u_{2p}u_{2m}\\ u_{1m}u_{2m}^{2}\\ u_{1p}u_{2p}\\ u_{1p}u_{2m}\\ u_{1m}u_{2p}\\ u_{1m}u_{2m}\\ u_{1p}^{2}u_{2p}\\ u_{1p}^{2}u_{2m}\\ u_{1p}u_{1m}u_{2p}\\ u_{1p}u_{1m}u_{2m}\\ u_{1m}^{2}u_{2p}\\ u_{1m}^{2}u_{2m}\\ u_{2p}^{3}\\ u_{2p}^{2}u_{2m}\\ u_{2p}u_{2m}^{2}\\ u_{2m}^{3}\\ u_{1p}^{2}\\ u_{1p}u_{1m}\\ u_{1m}^{2}\\ u_{2p}^{2}\\ u_{2p}u_{2m}\\ u_{2m}^{2} \end{array}\right]\;\mathrm{and}\;\beta \;\beta^{T}=\Omega^{2}\left[\begin{array}{cc} 8 & -\\ 0 & -\\ 0 & -\\ 8 & -\\ 0 & -\\ 0 & -\\ 8 & -\\ 0 & -\\ 0 & -\\ 8 & -\\ 3 & -\\ -1 & -\\ -1 & -\\ 3 & -\\ - & 8\\ - & 0\\ - & 0\\ - & 0\\ - & 0\\ - & 8\\ - & 8\\ - & 0\\ - & 0\\ - & 8\\ - & 3\\ - & -1\\ - & 3\\ - & 3\\ - & -1\\ - & 3 \end{array}\right],$$

where dashes in *β* represent elements whose values are of no significance as they correspond to elements in [*n*_*q*_] with a value of zero. Note that none of the nonlinear terms resulting from the effect of the static sag of the cable (described by *Q*) are resonant (i.e. correspond to a zero in *β*).

From equations (3.2), we may calculate [*n*_*u*_] (see equations (2.10)), and hence **N**_*u*_, using **N**_*u*_=[*n*_*u*_]u*. We may then calculate $N_{u,1}^{+}$ and $N_{u,2}^{+}$ (see equation (2.11)) as

3.3a $$N_{u,1}^{+}=\frac{W}{8}[3U_{1}^{3}\;e^{-j\phi_{1}}+2U_{1}U_{2}^{2}\;e^{-j\phi_{1}}+U_{1}U_{2}^{2}\;e^{+j(\phi_{1}-2\phi_{2})}]$$

and

3.3b $$N_{u,2}^{+}=\frac{W}{8}[3U_{2}^{3}\;e^{-j\phi_{2}}+2U_{1}^{2}U_{2}\;e^{-j\phi_{2}}+U_{1}^{2}U_{2}\;e^{-j(2\phi_{1}-\phi_{2})}].$$

Now, using equations (2.13) and (3.3), we may write

3.4a $$\left\{\omega_{n1}^{2}-\Omega^{2}+\frac{W}{4}[3U_{1}^{2}+2U_{2}^{2}+U_{2}^{2}\;e^{+j2(\phi_{1}-\phi_{2})}]\right\}U_{1}=0$$

and

3.4b $$\left\{\omega_{n2}^{2}-\Omega^{2}+\frac{W}{4}[3U_{2}^{2}+2U_{1}^{2}+U_{1}^{2}\;e^{-j2(\phi_{1}-\phi_{2})}]\right\}U_{2}=0,$$

where we have used *Ω*=*ω*_*r*1_=*ω*_*r*2_. The real parts of equations (3.4) then lead to

3.5a $$\left\{\omega_{n1}^{2}-\Omega^{2}+\frac{W}{4}[3U_{1}^{2}+(2+p)U_{2}^{2}]\right\}U_{1}=0$$

and

3.5b $$\left\{\omega_{n2}^{2}-\Omega^{2}+\frac{W}{4}[3U_{2}^{2}+(2+p)U_{1}^{2}]\right\}U_{2}=0,$$

where $p=\cos[2(\phi_{1}-\phi_{2})]$. The imaginary parts of equations (3.4) lead to $\sin[2(\phi_{1}-\phi_{2})]=0$ for both equations. As a result, *p*=±1 where *p*=+1 corresponds to responses where *u*_1_ and *u*_2_ are in-phase or anti-phase, and *p*=−1 corresponds to *u*_1_ and *u*_2_ being ±90° out-of-phase.

Besides the trivial solution where *U*_1_=0 and *U*_2_=0, corresponding to no motion, two solutions to equations (3.5) can be found by setting *U*_1_≠0 and *U*_2_=0, and *U*_1_=0 and *U*_2_≠0. These correspond to sets of responses of the system, denoted *S*1 and *S*2, which are defined by

3.6 $$\text{S}1:\;U_{2}=0,\;\Omega^{2}=\omega_{n1}^{2}+\frac{3W}{4}U_{1}^{2}$$

and

3.7 $$\text{S}2:\;U_{1}=0,\;\Omega^{2}=\omega_{n2}^{2}+\frac{3W}{4}U_{2}^{2}.$$

When both *U*_1_≠0 and *U*_2_≠0, equations (3.5) can be written as follows:

3.8 $$\Omega^{2}=\omega_{n1}^{2}+\frac{W}{4}[3U_{1}^{2}+(2+p)U_{2}^{2}]=\omega_{n2}^{2}+\frac{W}{4}[3U_{2}^{2}+(2+p)U_{1}^{2}],$$

which may be rearranged to give

3.9 $$\frac{W}{4}[(1-p)U_{1}^{2}+(p-1)U_{2}^{2}]=\omega_{n2}^{2}-\omega_{n1}^{2}.$$

As *ω*_*n*2_≠*ω*_*n*1_ (due to the influence of gravity), *p*=+1 cannot be a valid solution.

For the case where *p*=−1 two additional sets of responses, denoted *S*3^+^ and *S*3^−^ (or *S*3^±^ when referring to both), are produced. *S*3^±^ have the phase differences (*ϕ*_1_−*ϕ*_2_)=±*π*/2. Substituting *p*=−1 into equation (3.9) and rearranging gives the amplitude relationship

3.10a $$\text{S}3^{\pm }:\;\;\frac{W}{2}(U_{1}^{2}-U_{2}^{2})=\omega_{n2}^{2}-\omega_{n1}^{2},$$

and substituting this into equation (3.8) gives the response frequency relationship

3.10b $$\text{S}3^{\pm }:\;\Omega^{2}=\frac{1}{2}(\omega_{n1}^{2}+\omega_{n2}^{2})+\frac{W}{2}(U_{1}^{2}+U_{2}^{2}).$$

Owing to the ±*π*/2 phase differences, the responses represented by *S*3^±^ are out-of-unison resonances between *q*_1_ and *q*_2_. Physically, these are representative of a whirling in the first linear modes of the cable, where the two solutions represent clockwise and anticlockwise motions. The identical amplitude and frequency relationships for *S*3^+^ and *S*3^−^ illustrates that the behaviour of the cable is unaffected by the direction of motion. As the *S*3^±^ branches meet *S*1 (where *U*_2_=0) at a bifurcation, the point on *S*3^±^ where *U*_2_=0 is the point at which the bifurcation occurs. From equations (3.10), this point is described by

3.11 $$U_{1}^{2}=\frac{2}{W}(\omega_{n2}^{2}-\omega_{n1}^{2})\;\mathrm{and}\;\Omega^{2}=\frac{1}{2}(3\omega_{n2}^{2}-\omega_{n1}^{2}),$$

which is also a point on *S*1, which can be seen from equation (3.6). These results define the response in **u**. For the full response, the transform **q**=**u**+**h** may be used, in which **h** contains the harmonic contents of the response. Here we assume that **h** is negligible.

### (b) Example of a cable system

We now consider a cable with a length of 1.5 m, a diameter of 5 mm, a density of 3000 kg m^−3^, a Young's Modulus of 2×10^11^ Pa and a static tension of 200 N. The equations derived by Warnitchai et al. [32] lead to a system with linear natural frequencies *ω*_*n*1_=122.04 rad s^−1^ and *ω*_*n*2_=123.87 rad s^−1^, and nonlinear coefficient *W*=3.2×10^8^ m^−2^ s^−2^. The nonlinear coefficient *Q* (see equation (3.1)) is not needed as we do not consider any harmonic components.

**Figure 3.**
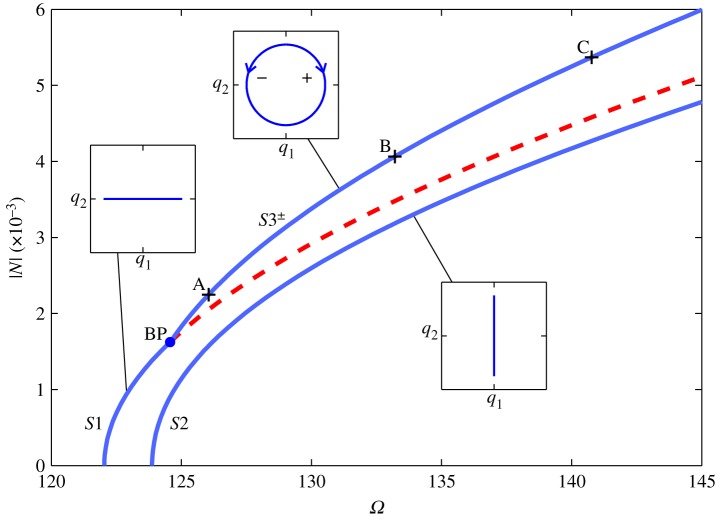
Responses of a cable with the parameter values *ω*_*n*1_=122.04 rad s^−1^, *ω*_*n*2_=123.87 rad s^−1^ and *W*=3.2×10^8^ m^−2^ s^−2^ in the projection of the common response frequency, *Ω*, against the absolute displacement amplitude, ∣N∣. The solid and dashed lines represent stable and unstable responses, respectively. A dot labelled ‘BP’ shows the pitchfork bifurcation from *S*1 onto *S*3^±^. Three embedded plots, in the projection of *q*_1_ against *q*_2_, illustrate the responses on the branches, parametrized in time. In the embedded plot showing out-of-unison motion, arrows (labelled ‘+’ and ‘−’) represent the clockwise and anticlockwise responses on *S*3^+^ and *S*3^−^, respectively. (Online version in colour.)

The responses of this cable, calculated using the numerical continuation software AUTO-07p [33], are shown in figure 3. This figure shows the projection of the common response frequency, *Ω*, against the absolute displacement amplitude $|N|=\sqrt{Q_{1}^{2}+Q_{2}^{2}}$, where *Q*_1_ and *Q*_2_ are the maximum amplitudes of *q*_1_ and *q*_2_, respectively. In the region shown here, the maximum error in ∣N∣, between the results of the continuation and the second-order normal form technique, is less than 3%. The predicted position of the bifurcation point (see equation (3.11)) is slightly different to the numerically computed position in both *Ω* and ∣N∣. A metric based on the Euclidean norm of the errors in *Ω* and ∣N∣ gives an error of 3.2%. These small errors validate both the accuracy of the results of the technique and the assumption that the harmonics are negligible (as the numerically computed results contain harmonics). These numerical results also determine the stability of the responses, although the stability of solutions may also be found analytically [30,34].

Three embedded plots in figure 3 illustrate the responses of the cable in the linear modal coordinates in the projection *q*_1_ against *q*_2_. The embedded plot showing out-of-unison motion is illustrative of responses on *S*3^+^ and *S*3^−^ for clockwise and anticlockwise motion, respectively (represented by opposing arrows labelled ‘+’ and ‘−’, respectively). As with figure 2*b*, the responses shown in the embedded plots are analogous to the paths of motion of the cable in the *y*–*z* plane. Three points labelled A, B and C on the *S*3^+^ branch correspond to the three responses depicted in figure 2. From figure 2*a* it can be seen that, in the projection shown in figure 2*b*, the cable is whirling in a clockwise direction. If the corresponding responses on *S*3^−^ were shown, the motion would be anticlockwise.

Although it is not shown here, the responses in *S*1 contain a small component of *q*_2_ responding at twice the frequency of *q*_1_—representative of a *swaying* motion of the cable. As this is small (*Q*_2_<0.01*Q*_1_ in the stable region) it does not violate the assumptions made in the normal form analysis and would be predicted as a harmonic response. The bifurcation on *S*1 shows the point at which the horizontal swaying motion loses stability and becomes a stable whirling motion (on *S*3^±^). This is a supercritical pitchfork bifurcation, with *S*1 losing stability and two stable branches, *S*3^±^, emerging. Owing to the projection used in figure 3, the *S*3^+^ and *S*3^−^ branches are superposed, as the amplitude of the whirling is unaffected by the direction of motion.

In this section, it has been shown, using the second-order normal form technique, that the whirling of a cable is an out-of-unison resonant response. In the next section, we show that out-of-unison resonance may also exist in in-line systems.

## 4. An in-line oscillator

We now consider an in-line, symmetric, 2-d.f. oscillator with forcing and damping (although we focus initially on the undamped case). This differs from the cable system considered in §3 insofar as it is non-conservative and the coordinates share the same physical dimension, *x*. To understand the underlying dynamics of this system, we consider its backbone curves. These describe the loci of responses of the unforced, undamped equivalent system, and they relate to the responses of the system when forced and damped.

**Figure 4.**
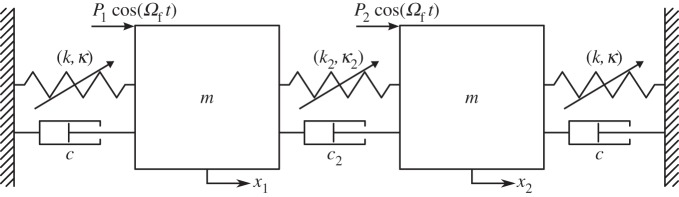
A schematic of an in-line, 2-d.f. oscillator with a symmetric structure. Identical cubic nonlinear springs, with linear spring constant *k* and cubic nonlinear constant *κ*, connect the masses to ground. Linear viscous dampers, with damping constant *c*, also ground the masses. Connecting the masses are a cubic nonlinear spring, with linear spring constant *k*_2_ and cubic nonlinear constant *κ*_2_, and a linear viscous damper, with damping constant *c*_2_. The displacements of the masses are written *x*_1_ and *x*_2_. Both masses are subjected to sinusoidal forcing at frequency *Ω*_*f*_ and amplitudes *P*_1_ and *P*_2_ as shown.

This system, shown in figure 4, is similar to that considered in [30]; however, here we consider a more general case where the springs may be hardening or softening, leading to additional solutions. Two lumped masses, both of mass *m*, have displacements *x*_1_ and *x*_2_ and are each forced sinusoidally at amplitudes *P*_1_ and *P*_2_, respectively, and at frequency *Ω*_*f*_. They are connected to ground via linear viscous dampers, damping constant *c*, and via nonlinear springs with the force–deflection relationship *F*=*k*(Δ*x*)+*κ*(Δ*x*)^3^. The masses are also connected via a linear viscous damper, constant *c*_2_, and a nonlinear spring with the force–deflection relationship *F*=*k*_2_(Δ*x*)+*κ*_2_(Δ*x*)^3^.

### (a) Backbone curves

The equations of motion of the forced and damped system shown in figure 4 can be written in the form of equation (2.1). However, as we are interested in the backbone curves of this system, we may describe the motion directly in the form of equation (2.2), with

4.1 $$\text{M}=\left[\begin{array}{cc} m & 0\\ 0 & m \end{array}\right],\;\text{K}=\left[\begin{array}{cc} k+k_{2} & -k_{2}\\ -k_{2} & k+k_{2} \end{array}\right]\;\mathrm{and}\;{\text{N}}_{x}=\left(\begin{array}{c} \kappa x_{1}^{3}+\kappa_{2}{\left(x_{1}-x_{2}\right)}^{3}\\ \kappa x_{2}^{3}+\kappa_{2}{\left(x_{2}-x_{1}\right)}^{3} \end{array}\right),$$

where *κ* and *κ*_2_ may be positive or negative. We may now apply the linear modal transform, **x**=*Φ***q**, to equation (4.1) such that it may be written in the form of equation (2.3), where

4.2 $$\Phi \;\Phi =\left[\begin{array}{cc} 1 & 1\\ 1 & -1 \end{array}\right],\;\Lambda \;\Lambda =\left[\begin{array}{cc} \omega_{n1}^{2} & 0\\ 0 & \omega_{n2}^{2} \end{array}\right]\;\mathrm{and}\;{\text{N}}_{q}=\frac{\kappa }{m}\left(\begin{array}{c} q_{1}^{3}+3q_{1}q_{2}^{2}\\ 3q_{1}^{2}q_{2}+\gamma q_{2}^{3} \end{array}\right),$$

where *γ*=1+8*κ*_2_/*κ*, and *ω*_*n*1_ and *ω*_*n*2_ are the first and second linear natural frequencies, respectively. These are calculated as $\omega_{n1}^{2}=k/m$ and $\omega_{n2}^{2}=(k+2k_{2})/m$. Here we assume that *ω*_*n*1_ and *ω*_*n*2_ are close, such that the fundamental components of *q*_1_ and *q*_2_ respond at the same frequency. We also assume that, when the system is forced near resonance, the fundamental components of *q*_1_ and *q*_2_ will respond at the forcing frequency *Ω*_*f*_. Hence, for convenience, we define the common response frequency as *Ω*, i.e. *Ω*=*Ω*_*f*_=*ω*_*r*1_=*ω*_*r*2_ where, as in §3, *ω*_*rn*_ is the fundamental response frequency of *q*_*n*_ for *n*=1,2. We now make the substitution *q*_*n*_=*u*_*n*_=*u*_*np*_+*u*_*nm*_ into **N**_*q*_, from which we calculate [*n*_*q*_], u* and *β* as

4.3 $${\left[n_{q}\right]}^{T}=\frac{\kappa }{m}\left[\begin{array}{cc} 1 & 0\\ 3 & 0\\ 3 & 0\\ 1 & 0\\ 3 & 0\\ 6 & 0\\ 3 & 0\\ 3 & 0\\ 6 & 0\\ 3 & 0\\ 0 & 3\\ 0 & 3\\ 0 & 6\\ 0 & 6\\ 0 & 3\\ 0 & 3\\ 0 & \gamma \\ 0 & 3\gamma \\ 0 & 3\gamma \\ 0 & \gamma \end{array}\right],\;{\text{u}}^{*}=\left[\begin{array}{c} u_{1p}^{3}\\ u_{1p}^{2}u_{1m}\\ u_{1p}u_{1m}^{2}\\ u_{1m}^{3}\\ u_{1p}u_{2p}^{2}\\ u_{1p}u_{2p}u_{2m}\\ u_{1p}u_{2m}^{2}\\ u_{1m}u_{2p}^{2}\\ u_{1m}u_{2p}u_{2m}\\ u_{1m}u_{2m}^{2}\\ u_{1p}^{2}u_{2p}\\ u_{1p}^{2}u_{2m}\\ u_{1p}u_{1m}u_{2p}\\ u_{1p}u_{1m}u_{2m}\\ u_{1m}^{2}u_{2p}\\ u_{1m}^{2}u_{2m}\\ u_{2p}^{3}\\ u_{2p}^{2}u_{2m}\\ u_{2p}u_{2m}^{2}\\ u_{2m}^{3} \end{array}\right]\;\mathrm{and}\;\beta \;\beta^{T}=\Omega^{2}\left[\begin{array}{cc} 8 & -\\ 0 & -\\ 0 & -\\ 8 & -\\ 8 & -\\ 0 & -\\ 0 & -\\ 0 & -\\ 0 & -\\ 8 & -\\ - & 8\\ - & 0\\ - & 0\\ - & 0\\ - & 0\\ - & 8\\ - & 8\\ - & 0\\ - & 0\\ - & 8 \end{array}\right],$$

where elements in *β* containing a dash correspond to zero-valued elements in [*n*_*q*_].

We may now calculate [*n*_*u*_] using equations (2.10) and (4.3). The relationship **N**_*u*_=[*n*_*u*_]u* may then be used to find $N_{u,1}^{+}$ and $N_{u,2}^{+}$ (see equation (2.11)) as

4.4a $$N_{u,1}^{+}=\frac{3\kappa }{8m}[U_{1}^{3}\;e^{-j\phi_{1}}+2U_{1}U_{2}^{2}\;e^{-j\phi_{1}}+U_{1}U_{2}^{2}\;e^{+j(\phi_{1}-2\phi_{2})}]$$

and

4.4b $$N_{u,2}^{+}=\frac{3\kappa }{8m}[\gamma U_{2}^{3}\;e^{-j\phi_{2}}+2U_{1}^{2}U_{2}\;e^{-j\phi_{2}}+U_{1}^{2}U_{2}\;e^{-j(2\phi_{1}-\phi_{2})}].$$

Now, using equations (2.13) and (4.4), we may write

4.5a $$\left[\omega_{n1}^{2}-\Omega^{2}+\frac{3\kappa }{4m}\{U_{1}^{2}+U_{2}^{2}(2+e^{+j2(\phi_{1}-\phi_{2})})\}\right]U_{1}=0$$

and

4.5b $$\left[\omega_{n2}^{2}-\Omega^{2}+\frac{3\kappa }{4m}\{\gamma U_{2}^{2}+U_{1}^{2}(2+e^{-j2(\phi_{1}-\phi_{2})})\}\right]U_{2}=0.$$

The two solutions in which *u*_1_ and *u*_2_ are independently non-zero are labelled *S*1 and *S*2, respectively

4.6 $$\text{S}1:\;U_{2}=0,\;\Omega^{2}=\omega_{n1}^{2}+\frac{3\kappa }{4m}U_{1}^{2}$$

and

4.7 $$\text{S}2:\;U_{1}=0,\;\Omega^{2}=\omega_{n2}^{2}+\frac{3\kappa \gamma }{4m}U_{2}^{2}.$$

When *u*_1_ and *u*_2_ are both non-zero, equations (4.5) may be written as follows:

4.8 $$\Omega^{2}=\omega_{n1}^{2}+\frac{3\kappa }{4m}\{U_{1}^{2}+U_{2}^{2}(2+p)\}=\omega_{n2}^{2}+\frac{3\kappa }{4m}\{\gamma U_{2}^{2}+U_{1}^{2}(2+p)\},$$

where, as equation (4.8) must be real, the phase difference is given by

4.9 $$p=e^{j2|\phi_{1}-\phi_{2}|}=\pm 1,$$

such that *p*=+1 corresponds to $|\phi_{1}-\phi_{2}|=0,\pi$ (i.e. *u*_1_ and *u*_2_ are in-phase or anti-phase), and *p*=−1 corresponds to $|\phi_{1}-\phi_{2}|=\pi /2$ (i.e. *u*_1_ and *u*_2_ are ±90° out-of-phase). Setting *p*=+1 yields two backbone curves, labelled *S*3^+^ and *S*3^−^, with the phase differences

4.10 $$S3^{+}:\;|\phi_{1}-\phi_{2}|=0\;\mathrm{and}\;S3^{-}:\;|\phi_{1}-\phi_{2}|=\pi .$$

Substituting *p*=+1 into equation (4.8) leads to the amplitude and response frequency relationships

4.11a $$\text{S}3^{\pm }:\;\frac{3\kappa }{2m}(U_{2}^{2}-U_{1}^{2})-\frac{6\kappa_{2}}{m}U_{2}^{2}=\omega_{n2}^{2}-\omega_{n1}^{2}$$

and

4.11b $$\text{S}3^{\pm }:\;\Omega^{2}=\omega_{n1}^{2}+\frac{3\kappa }{4m}(U_{1}^{2}-U_{2}^{2})+\frac{3\kappa }{m}U_{2}^{2}.$$

The case where *p*=−1 yields a further two backbone curves denoted *S*4^+^ and *S*4^−^. These are characterized by the phase differences

4.12 $$\text{S}4^{+}:\;\phi_{1}-\phi_{2}=+\frac{\pi }{2}\;\mathrm{and}\;\text{S}4^{-}:\;\phi_{1}-\phi_{2}=-\frac{\pi }{2}.$$

Substituting *p*=−1 into equation (4.8) gives the amplitude and response frequency relationships

4.13a $$\text{S}4^{\pm }:\;\frac{-6\kappa_{2}}{m}U_{2}^{2}=\omega_{n2}^{2}-\omega_{n1}^{2}$$

and

4.13b $$\text{S}4^{\pm }:\;\Omega^{2}=\omega_{n1}^{2}+\frac{3\kappa }{4m}(U_{1}^{2}+U_{2}^{2}).$$

From the phase relationships given in equation (4.12), it can be seen that, for this system, responses on the *S*4^±^ branches are out-of-unison.

**Figure 5.**
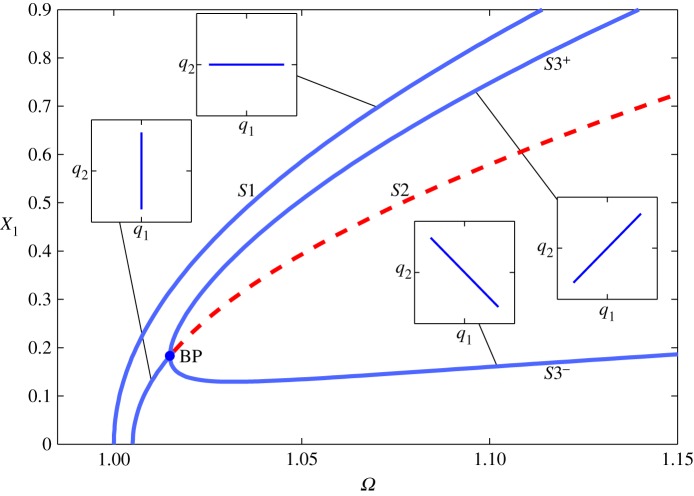
Backbone curves for the system represented in figure 4 with the parameter values *m*=1, *ω*_*n*1_=1, *ω*_*n*2_=1.005, *κ*=0.4 and *κ*_2_=0.05. The responses are shown in the projection of common response frequency, *Ω*, against the maximum amplitude of displacement of the first physical coordinate *X*_1_. Solid and dashed lines represent stable and unstable responses, respectively. The symmetry-breaking pitchfork bifurcation from *S*2 onto *S*3^±^ is indicated with a dot and labelled ‘BP’. Four embedded plots, in the projection of *q*_1_ against *q*_2_, illustrate the responses on the backbone curves, parametrized in time. (Online version in colour.)

As an example, we now briefly consider the system described in [30], in which the springs are hardening. Figure 5 shows the backbone curves *S*1, *S*2 and *S*3^±^ for this system, in the projection of the common response frequency, *Ω*, against the maximum amplitude of displacement of the first physical coordinate, *X*_1_. As in the previous example, these results were calculated using numerical continuation and are in good agreement with the results of the second-order normal form technique. The maximum error between these methods in *X*_1_ is less than 1% and the error in the position of the bifurcation point, measured as the Euclidean norm of the errors in *Ω* and *X*_1_, is less than 0.03%. Four embedded plots illustrate the responses on the backbone curves in the projection *q*_1_ against *q*_2_, parametrized in time. Equations (4.11) show that *S*3^±^ are composed of both *q*_1_ and *q*_2_, giving asymmetric responses in *x*_1_ and *x*_2_. This asymmetry is such that figure 5 would appear to be identical if shown in the projection *Ω* against *X*_2_ (rather than *X*_1_), except that *S*3^+^ and *S*3^−^ would be interchanged. For further details, see [30].

For this system, with these parameters, out-of-unison responses are not seen (i.e. there are no valid solutions for *S*4^±^). To investigate why *S*4^±^ do not exist here, we now consider how the nonlinear parameters, *κ* and *κ*_2_, affect the existence of the backbone curves.

### (b) Effects of nonlinear parameters on the existence of backbone curves

We now investigate the ranges of values of the nonlinear parameters, *κ* and *κ*_2_, for which the backbone curves *S*3^±^ and *S*4^±^ may exist. This is done by firstly finding the parameter values that yield valid solutions to the bifurcations onto *S*3^±^ and *S*4^±^, as a valid bifurcation must lead to a valid backbone curve. We then investigate whether the backbone curves may exist without a bifurcation, i.e. whether the ranges that yield non-physically valid bifurcations may lead to physically valid *S*3^±^ and *S*4^±^ solutions. We define a valid solution as one in which the common response frequency, *Ω*, and amplitudes, *U*_*n*_, are real and positive. We assume that *m*>0 and *ω*_*n*2_>*ω*_*n*1_.

Equation (4.13a) shows that *U*_2_=0 cannot be a solution on *S*4^±^. Therefore, *S*4^±^ cannot meet *S*1 at any point. Using equations (4.13), the point at which *U*_1_=0 on *S*4^±^ is given by

4.14a $$U_{2}^{2}=\frac{m}{6\kappa_{2}}(\omega_{n1}^{2}-\omega_{n2}^{2})$$

and

4.14b $$\Omega^{2}=\frac{\kappa }{8\kappa_{2}}(\gamma \omega_{n1}^{2}-\omega_{n2}^{2}).$$

Equation (4.7) shows that this is a solution for *S*2, and hence represents the position of the bifurcation from *S*2 onto *S*4^±^. Using equations (4.14), and recalling that *γ*=1+8*κ*_2_/*κ*, we find the relationships

4.15a $$\kappa_{2}<0\;\text{if}:\;\kappa >0$$

and

4.15b $$\kappa_{2}<\frac{\kappa }{8}\left(\frac{\omega_{n2}^{2}}{\omega_{n1}^{2}}-1\right)\;\text{if}:\;\kappa <0,$$

which must be satisfied for the existence of valid solutions of the bifurcation onto *S*4^±^. It may be possible for valid solutions to exist that originate from a non-real bifurcation point. To investigate this, from equations (4.15), the regions in which the bifurcation onto *S*4^±^ does not exist are given by

4.16a $$\kappa_{2}>0\;\text{if:}\;\kappa >0$$

and

4.16b $$\frac{\kappa }{8}\left(\frac{\omega_{n2}^{2}}{\omega_{n1}^{2}}-1\right)-\kappa_{2}<0\;\text{if:}\;\kappa <0.$$

Using equation (4.13a), it can be seen that *κ*_2_>0 cannot lead to any valid *S*4^±^ solution, regardless of the value of *κ*. Thus, the region defined by equation (4.16a) cannot yield an *S*4^±^ solution. To investigate the region given by equation (4.16b), we consider equation (4.13b) which, after eliminating *U*_2_ using equation (4.13a), may be written as follows:

4.17 $$\Omega^{2}=\left\{-\frac{\omega_{n1}^{2}}{\kappa_{2}}\left[\frac{\kappa }{8}\left(\frac{\omega_{n2}^{2}}{\omega_{n1}^{2}}-1\right)-\kappa_{2}\right]\right\}+\frac{3\kappa }{4m}U_{1}^{2}.$$

When equation (4.16b) is true, the expression within the square brackets in equation (4.17) is negative. As we have also determined that *κ*_2_<0 is required for a valid solution, the expression within the braces must be negative. As *κ*<0 (a condition given by equation (4.16b)), the final term in equation (4.17) is also negative, hence the right-hand side of the equation is negative and no valid solution exists. Therefore, equations (4.15) describe the only regions in which solutions to *S*4^±^ may exist.

**Figure 6.**
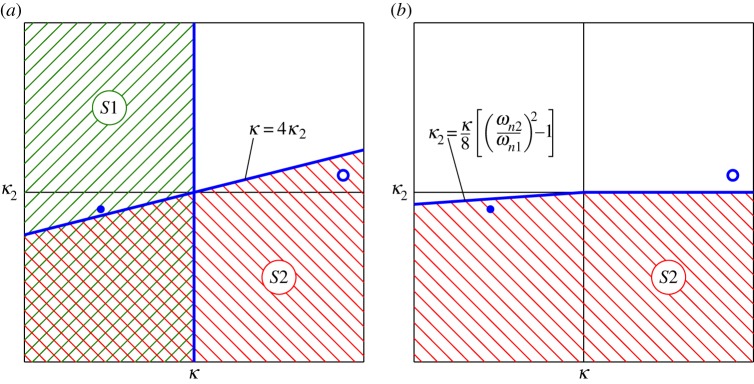
Graphical representations of the regions in which there exists solutions for the *S*3^±^ and *S*4^±^ backbone curves in panels (*a*) and (*b*), respectively. These are in the projection of *κ* against *κ*_2_. Hatched-green areas represent regions where a backbone curve bifurcation exists on *S*1, hatched-red areas show where a bifurcation exists on *S*2 and cross-hatched green and red areas show where there are bifurcations on both *S*1 and *S*2. Thin-black lines represent *κ*=0 and *κ*_2_=0. Thick-blue lines show the boundaries of the regions. A blue circle and a blue dot represent the positions of the systems used in figure 5 and in figure 7, respectively.

**Figure 7.**
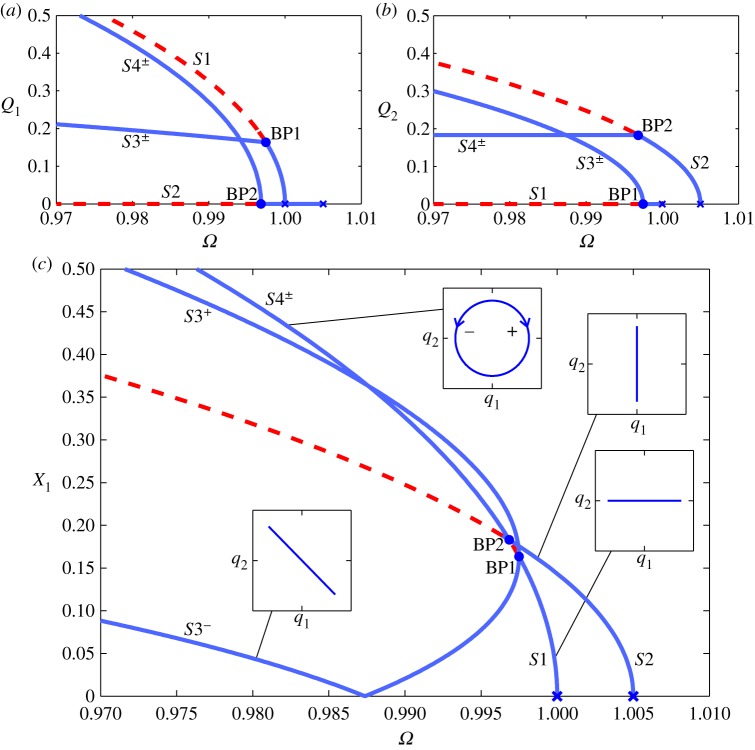
Backbone curves for a system where *m*=1, *ω*_*n*1_=1, *ω*_*n*2_=1.005, *κ*=−0.25 and *κ*_2_=−0.05. The responses are shown in the projection of common response frequency, *Ω*, against the maximum amplitude of displacement of the first linear modal coordinate (*Q*_1_) in (*a*), the second linear modal coordinate (*Q*_2_) in (*b*) and the first physical coordinate (*X*_1_) in (*c*). Solid-blue and dashed-red lines represent stable and unstable responses, respectively. Hamiltonian Hopf bifurcations from the trivial, zero-amplitude, response onto *S*1 and *S*2 are indicated with blue crosses. The symmetry-breaking pitchfork bifurcations from *S*1 onto *S*3^±^ and *S*2 onto *S*4^±^ are represented with blue dots labelled ‘BP1’ and ‘BP2’, respectively. In (*c*), four embedded plots, in the projection of *q*_1_ against *q*_2_, illustrate the responses on the backbone curves, parametrized in time. In the embedded plot showing out-of-unison motion, arrows (labelled ‘+’ and ‘−’) represent the clockwise and anticlockwise responses (in this projection) on *S*4^+^ and *S*4^−^, respectively.

Using this approach, the position of the bifurcations onto *S*3^±^ can also be found and the existence of *S*3^±^ may be determined. This reveals that *S*3^±^ can emerge from both *S*1 and *S*2, and that no valid solution for *S*3^±^ exists when a valid bifurcation does not exist. The regions in which valid solutions for *S*3^±^ and *S*4^±^ exist are summarized in figure 6. The hatched-green and hatched-red areas show where bifurcations exist on *S*1 and *S*2, respectively. Thick-blue lines represent the boundaries of the regions. A blue circle and a blue dot represent the positions of the systems used in figure 5 and in figure 7, respectively. In figure 6*a*, the area cross-hatched with both green and red represents the region in which the *S*3^±^ backbone curves meet both *S*1 and *S*2. In figure 6*b*, it can be seen that the blue circle is outside any valid region, which is representative of no valid solutions to *S*4^±^ existing for the parameter values used in figure 5.

### (c) Out-of-unison motion in an in-line system

Figure 7 shows the backbone curves for a system where *m*=1, *ω*_*n*1_=1, *ω*_*n*2_=1.005, *κ*=−0.25 and *κ*_2_=−0.05. These are shown in the projection of the common response frequency, *Ω*, against the maximum amplitude of displacement of the first linear modal coordinate (*Q*_1_) in 7*a*, the second linear modal coordinate (*Q*_2_) in 7*b* and the first physical coordinate (*X*_1_) in 7*c*.

As illustrated by the blue dots in figure 6, backbone curves *S*3^±^ and *S*4^±^ all exist and bifurcate from *S*1 and *S*2, respectively. As with the example in figure 5, these are symmetry-breaking pitchfork bifurcations. The results shown here were calculated using numerical continuation and show good agreement with the predictions of the second-order normal form technique (not shown); specifically, the maximum error in *X*_1_ is less than 1%. The Euclidean norm of the error in *Ω* and *X*_1_ of the bifurcation positions is less than 0.001% for the bifurcation from *S*1 onto *S*3^±^ and less than 0.002% for the bifurcation from *S*2 onto *S*4^±^.

The bifurcation from *S*1 onto *S*3^±^ is indicated with a blue dot labelled ‘BP1’. It can be seen in figure 7*a,b* that this causes a loss of stability of the *S*1 branch for responses at amplitudes above this bifurcation. These also show that *S*3^±^ contain contributions from both *q*_1_ and *q*_2_, indicating an internal resonance between the two linear modal coordinates. Figure 7*c* shows *S*3^−^ reaching an amplitude of zero in the physical coordinate *X*_1_ at a particular response frequency *Ω*. As *q*_1_ and *q*_2_ are in anti-phase (see equation (4.10)) and *x*_1_=*q*_1_+*q*_2_ (see equation (4.2)) this is representative of the point at which *Q*_1_=*Q*_2_. From equations (4.11), it can be seen that, neglecting harmonics, this point occurs when $\Omega^{2}=\omega_{n1}^{2}+(\kappa /2\kappa_{2})(\omega_{n1}^{2}-\omega_{n2}^{2})$.

The bifurcation from *S*2 onto *S*4^±^ is represented with a blue dot labelled ‘BP2’. In all projections shown here, *S*4^+^ and *S*4^−^ are superposed. This is because, neglecting harmonics, the linear modal displacement amplitudes are identical in *S*4^+^ and *S*4^−^ (see equations (4.13)) and the phase differences, ±*π*/2, lead to identical displacement amplitudes in the physical coordinates. It can be seen in figure 7*b* that *Q*_2_ is constant on *S*4^±^, as predicted in equation (4.13a). As in the previous example, figure 7*c* would appear to be identical if shown in the projection *Ω* against *X*_2_, except that *S*3^+^ and *S*3^−^ would be interchanged (while *S*4^+^ and *S*4^−^ would remain superposed).

As the *S*4^±^ branches are stable, we see that the conservative in-line system represented by the backbone curves exhibits a set of stable responses, where the underlying linear modal coordinates are ±90° out-of-phase. This motion is similar to the whirling seen in the cables except that, in this system, the linear modal coordinates share the same spatial dimension. This demonstrates that two coordinates may resonate out-of-unison in an in-line system.

An out-of-unison response in the linear modal coordinates (*q*_1_ and *q*_2_) corresponds to a set of responses in the physical coordinates (*x*_1_ and *x*_2_) that are nearly out-of-unison. To find the point at which the physical coordinates are precisely out-of-unison, we use equation (4.2) such that (neglecting harmonics) we may write *x*_1_ and *x*_2_ as

4.18a $$x_{1}=u_{1}+u_{2}=U_{1}\cos(\Omega t-\phi_{1})+U_{2}\cos(\Omega t-\phi_{2})$$

and

4.18b $$x_{2}=u_{1}-u_{2}=U_{1}\cos(\Omega t-\phi_{1})-U_{2}\cos(\Omega t-\phi_{2}).$$

When *ϕ*_1_−*ϕ*_2_=±*π*/2, equations (4.18) may be written as follows:

4.19a $$x_{1}=U_{1}\cos(\Omega t-\phi_{1})\mp U_{2}\sin(\Omega t-\phi_{1})$$

and

4.19b $$x_{2}=U_{1}\cos(\Omega t-\phi_{1})\pm U_{2}\sin(\Omega t-\phi_{1}).$$

Letting $U_{1}=\tilde{U}\cos(\psi )$ and $U_{2}=\tilde{U}\sin(\psi )$, equations (4.19) can then be written

4.20a $$x_{1}=\tilde{U}\cos(\Omega t-\phi_{1}\pm \psi ),$$

and

4.20b $$x_{2}=\tilde{U}\cos(\Omega t-\phi_{1}\mp \psi ).$$

We may also write

4.21 $$\tan(\psi )=\frac{U_{2}}{U_{1}}.$$

When the phase difference between *x*_1_ and *x*_2_ is ±*π*/2 then, from equations (4.20), *ψ*=*π*/4. Substituting this into equation (4.21) leads to *U*_1_=*U*_2_. Hence, for responses on the *S*4^±^ branches (where the underlying linear modal coordinates are out-of-unison), if the amplitudes of the two linear modal coordinates are equal, the physical coordinates, *x*_1_ and *x*_2_, are also vibrating out-of-unison.

### (d) Forced response of an example system

**Figure 8.**
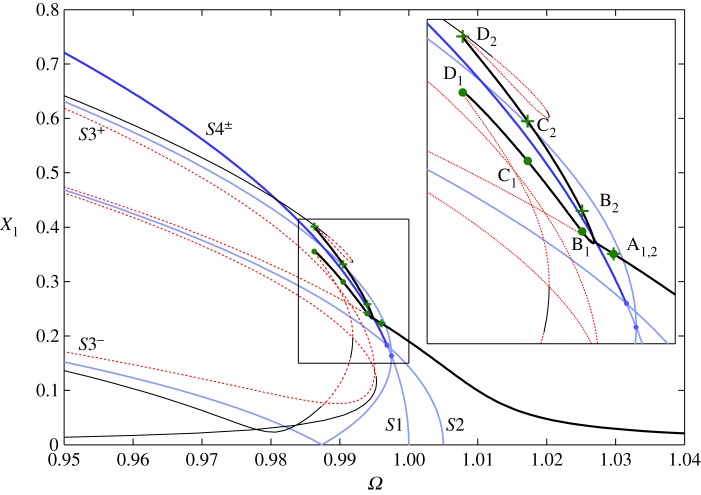
Backbone curves and forced, damped responses for the 2-d.f. in-line system with the parameters *m*=1, *ω*_*n*1_=1, *ω*_*n*2_=1.005, *κ*=−0.25, *κ*_2_=−0.05, *c*=0.002, *c*_2_=5×10^−6^ and [*P*_1_, *P*_2_]=[0.0015,−0.0015]. The responses are shown in the projection of common response frequency, *Ω*, against the maximum displacement amplitude of the first physical coordinate, *X*_1_. *S*1, *S*2 and *S*3^±^ are represented with light-blue lines, while *S*4^±^ are represented by dark-blue lines (superposed). The thin-black and dashed-red lines show the stable and unstable sections of the forced responses, respectively. The forced branch following *S*4^±^ is highlighted with a thick-black line and this section is shown in detail. Four sets of green dots and crosses labelled $A_{1,2}\to D_{1,2}$ correspond to four separate responses of the system—shown in figure 9, parametrized in time.

We now compare the backbone curves to the forced and damped responses of this system. The damping and forcing parameters used here are *c*=0.002, *c*_2_=5×10^−6^ and [*P*_1_,*P*_2_]=[0.0015,−0.0015]. All other system parameters are those used in §4*c*. Owing to the antisymmetric shape of the forcing, only the second underlying linear mode, *q*_2_, experiences direct modal forcing. We therefore expect the forced responses to only follow backbone curves with a *q*_2_ component (i.e. the responses will not follow *S*1). Figure 8 shows the backbone curves of this system (as presented in figure 7) compared with the forced responses. This is shown in the projection of the common response frequency, *Ω* (assumed to be equal to the forcing frequency, *Ω*_*f*_), against the maximum displacement amplitude of the first physical coordinate, *X*_1_. Light-blue lines represent *S*1, *S*2 and *S*3^±^ whilst dark-blue lines represent *S*4^±^ (which are superposed, as described in §4*c*). Thin-black and dashed-red lines show the stable and unstable forced responses, respectively. The forced responses that are of interest here (following *S*4^±^) are highlighted with a thick-black line. Other responses in figure 8 are beyond the scope of this study.

**Figure 9.**
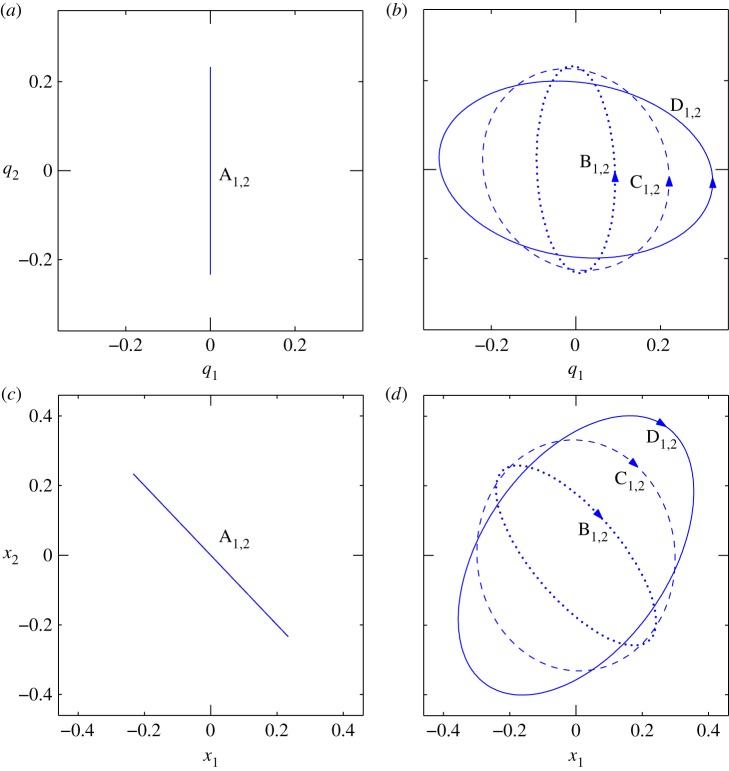
Four forced responses, parametrized in time, of the 2-d.f. in-line system with the parameters *m*=1, *ω*_*n*1_=1, *ω*_*n*2_=1.005, *κ*=−0.25, *κ*_2_=−0.05, *c*=0.002, *c*_2_=5×10^−6^ and [*P*_1_,*P*_2_]=[0.0015,−0.0015]. These responses correspond to the four sets of points in figure 8, $A_{1,2}\to D_{1,2}$, that are on the branch of forced responses following *S*4^−^. (*a*,*b*) Show the responses in the linear modal coordinates, *q*_1_ against *q*_2_, and (*c*,*d*) show the responses in the physical coordinates, *x*_1_ against *x*_2_. (*a*,*c*) Show that response A_1,2_ is composed only of *q*_2_ (as with *S*2). (*b*,*d*) Show that responses $B_{1,2}\to D_{1,2}$ are out-of-unison (as with *S*4^−^) aside from a slight distortion due to forcing and damping. Arrows in (*b*,*d*) show the direction of motion. (Online version in colour.)

As the excitation is in the shape of the second linear mode, there exists a typical Duffing-like softening response that envelops the backbone curve *S*2. This branch of the forced response (shown in figure 8) is referred to as the *primary branch* and, as with *S*2, is composed only of a response in *q*_2_. Near the frequency *Ω*=0.995, there is a bifurcation from this primary branch onto two *secondary branches* that follow *S*4^±^. These branches are internally resonant responses as *q*_1_ is present, but not directly forced. For amplitudes above this bifurcation, the primary branch becomes unstable. This is clearly analogous to the responses of the backbone curves, where *S*2 loses stability at amplitudes above the bifurcation onto *S*4^±^. At the points labelled D_1,2_ in figure 8, the secondary branches reach fold bifurcations and begin to follow *S*3^±^. As with the backbone curves, the symmetry of the system allows the physical coordinates to follow either of the secondary branches of the forced responses.

Four sets of points—labelled A_1,2_, B_1,2_, C_1,2_ and D_1,2_—represent four forced responses, where the subscripts 1 and 2 denote the responses in the coordinates *x*_1_ and *x*_2_, respectively. These responses, parametrized in time, are shown in figure 9, where figure 9*a,b* shows the responses in the linear modal coordinates, *q*_1_ against *q*_2_, and figure 9*c,d* shows the responses in the physical coordinates, *x*_1_ against *x*_2_. Response A_1,2_, on the primary forced branch, is shown in figure 9*a,c* and is clearly only composed of *q*_2_, which is represented in the physical coordinates by an oscillation in anti-phase. Responses $B_{1,2}\to D_{1,2}$ are situated on the secondary forced branch. Owing to the symmetry-breaking bifurcation, we can determine that this branch is tending towards the *S*4^−^ backbone curve. Responses $B_{1,2}\to D_{1,2}$ are represented in figure 9*b,d* and clearly demonstrate out-of-unison motion in the modal coordinates. A slight distortion, owing to the influence of the forcing and damping, is seen in the out-of-unison responses and the degree of distortion increases as the responses diverge from *S*4^−^ (i.e. the distortion in D_1,2_ is greater than in B_1,2_). Reducing the forcing and damping would result in the secondary branches collapsing onto *S*4^−^ and a decrease in the distortion of the responses.

Figure 9*b* shows that, aside from the distortion, the linear modal coordinates are out-of-unison for responses on the secondary branches, i.e. as one coordinate reaches an extrema the other passes through zero. Figure 9*d* shows that, in responses B_1,2_ and D_1,2_, the physical coordinates are neither in-unison nor out-of-unison, i.e. as one coordinate reaches an extrema or zero the other does not. In response C_1,2_, however, the physical coordinates are out-of-unison (aside from the distortion). As discussed in §4*c*, this corresponds to the point on the backbone curves where the amplitudes of *q*_1_ and *q*_2_ are equal (neglecting harmonics). The similarity between the corresponding responses in the forced system and in the backbone curves shows that, for lightly damped systems, the forced response is governed by the backbone curves.

## 5. Conclusion

In this paper, we have investigated the phenomenon of out-of-unison resonance for systems of weakly nonlinear, coupled oscillators. This phenomenon has physical manifestations, such as whirling in a taut cable, and we have shown how this phenomenon can be modelled in order to describe the dynamic behaviour of the system. A cable was modelled using two sets of modes, one in the vertical and the other in the transverse direction, where the natural frequencies of the corresponding modes are close. The proximity of these natural frequencies allows for resonant interactions between the modes at a 1 : 1 ratio and leading to out-of-unison resonance. We then investigated an in-line nonlinear oscillator, with two close linear natural frequencies. Using a backbone curve analysis, we demonstrated that there were specific parameters for this system that led to out-of-unison resonance. In addition to this, we considered the case where the system was forced and lightly damped using continuation methods to track the steady-state periodic motion. As would be expected for a lightly damped system, the forced responses were shown to be governed by the backbone curves defined for the underlying conservative system. Therefore, for this particular system, we were able to show examples of resonances in the forced responses that have similar features to those obtained from studying the underlying conservative system.
